# PIGS: improved estimates of identity-by-descent probabilities by probabilistic IBD graph sampling

**DOI:** 10.1186/1471-2105-16-S5-S9

**Published:** 2015-03-18

**Authors:** Danny S Park, Yael Baran, Farhad Hormozdiari, Celeste Eng, Dara G Torgerson, Esteban G Burchard, Noah Zaitlen

**Affiliations:** 1 Department of Bioengineering and Therapeutic Sciences, University of California San Francisco, San Francisco, CA, USA; 2 Department of Computer Science, Tel Aviv University, Tel Aviv, Israel; 3 Department of Computer Science, University of California Los Angeles, 90095 Los Angeles, CA, USA; 4 Department of Medicine, University of California San Francisco, 94158 San Francisco, CA, USA

**Keywords:** Identity-by-Descent, Graph Sampling, Probabilistic Graph

## Abstract

Identifying segments in the genome of different individuals that are identical-by-descent (IBD) is a fundamental element of genetics. IBD data is used for numerous applications including demographic inference, heritability estimation, and mapping disease loci. Simultaneous detection of IBD over multiple haplotypes has proven to be computationally difficult. To overcome this, many state of the art methods estimate the probability of IBD between each pair of haplotypes separately. While computationally efficient, these methods fail to leverage the clique structure of IBD resulting in less powerful IBD identification, especially for small IBD segments.

We develop a hybrid approach (PIGS), which combines the computational efficiency of pairwise methods with the power of multiway methods. It leverages the IBD graph structure to compute the probability of IBD conditional on all pairwise estimates simultaneously. We show via extensive simulations and analysis of real data that our method produces a substantial increase in the number of identified small IBD segments.

## Background

Identity-by-descent (IBD) is a fundamental genetics concept with broad applications to both medical and population genetics [1]. Two haplotypes are identical-by-state (IBS) if they share the same sequence. Two haplotypes are IBD if they are both IBS and were inherited from a common ancestor [2]. IBD therefore contains information both about sequence similarity and about the historical relationship of individuals. IBD has been used for such applications as detecting cryptic relatedness between individuals [3], estimating components of heritability [4], inferring evolutionary and demographic history [5-7], and mapping disease loci [8-13]. Therefore, the identification of IBD segments from genome-wide genotyping studies, and more recently sequencing studies, has important implications for studies of complex human phenotypes.

The identification of IBD segments is challenging for both statistical and computational reasons. IBD segments may be missed due to genotyping or sequencing errors. Since IBD occurs at the level of haplotypes, the data are typically phased and phasing errors can induce false negatives. Small segments of IBD are especially challenging because their haplotype frequency must be accurately modeled often resulting in both false positive and false negative IBD calls [14]. Finally, there are computational challenges because the number of potential IBD relationships at a locus is O(2^*h*(*h *− 1)/2^), where *h *is the number of haplotypes.

Two classes of methods for computing the probability of IBD between haplotypes have been developed. Multiway methods such as Moltke *et al*. 2011[15], simultaneously estimate the probability of IBD over the haplotypes of all individuals in a study. While powerful, generally multiway approaches are not computationally efficient enough to examine whole genome data sets over a large number of individuals [16]. Recently an efficient mulitway method, HapFABIA [7], has been proposed but focuses on detecting very ancient IBD segments (i.e. <<1 cM) and relies on the existence of rare variantion in the data. In practice, pairwise methods such as Germline [17] and Refined IBD [14] are used to detect segments of IBD between pairs of haplotypes independently. Germline uses a sliding-window dictionary approach to find putative IBD segments and relies on fragment length to estimate IBD probability. Refined IBD utilizes the Germline approach to identify putative IBD segments and then applies a hidden markov model (HMM) to compute haplotype frequencies and estimate IBD probabilities. Since these methods consider pairs of individuals independently, they are computationally efficient at the genome-wide scale. However, they do not exploit the clique structure of true IBD segments [12,18], and lack power relative to multiway approaches for smaller IBD segments [15,18,7].

Here we introduce a novel method PIGS, which combines the computational efficiency of pairwise methods with the power of multiway methods. PIGS takes as input the IBD probabilities output by pairwise approaches. Then, to update the probability that a pair of haplotypes are IBD, it computes the probability of IBD conditional on the IBD probabilities of all other haplotypes pairs at the locus. Consider a pair of haplotypes with a low probability of being IBD according to a pairwise method. If both haplotypes are IBD with high probability to a shared set of many other haplotypes, then that pair has a higher probability of being IBD conditioned on the shared set. By leveraging the graph structure of the complete set of IBD segments we are able to produce more accurate estimate of IBD probabilities and thereby produce more powerful identification of IBD segments. We first present an exact algorithm for computing conditional IBD probabilities. However, because of the large number of potential IBD graphs we can not compute exact probabilities in all cases. Instead, we propose an efficient sampling algorithm to approximate these probabilities in practice.

Once IBD probabilities are computed from PIGS or other methods such as Germline and Refined IBD, IBD status can be *called *by several approaches. In thresholding approaches, all segments exceeding a probability cutoff are output as IBD. Recent clique-calling methods such as DASH [12], IBD-Groupon [18], and Efficient Multiple-IBD (EMI) algorithm [19] have been proposed to output cliques of IBD. The probabilities output by PIGS can be used as input to either thresholding or clique-based methods and we discuss the relative merits of these approaches in detail below (see Results). We show via extensive simulations and real data analysis that IBD called from a simple thresholding approach to PIGS output outperforms all previous efficient approaches on several metrics. We observed a 95% increase in the total number of identified IBD segments of 0.5 centimorgans and a 40% increase in identified IBD segments across all sizes with only a modest increase in error rate. Finally we show that the clique-calling methods DASH and EMI are more powerful when using the output of PIGS compared to Refined IBD, with increases in power ranging from 1% to 5%.

## Methods

### IBD graph

An IBD graph is constructed over a set of N haplotypes at a genomic locus as follows. Each haplotype is represented by a node and there exists an edge between nodes if the two haplotypes to which the nodes correspond are IBD at the locus. Valid IBD graphs obey a transitivity property such that if individuals 1 and 2 are IBD and individuals 2 and 3 are IBD, then individuals 1 and 3 are IBD [20]. An IBD graph is *transitive *if the edges obey the transitivity property, otherwise the graph is *intransitive *and can not represent the true state of IBD at the locus. Due to the transitivity property, all connected components of a valid IBD graph at a locus are cliques. We leverage the clique property of IBD graphs to improve the pairwise probabilities of IBD output by existing software packages such as Refined IBD [14].

### Probabilistic IBD graph

Given the probability of IBD between all pairs of *N *haplotypes at a genomic locus, we construct a probabilistic IBD graph *G_P _*= (N, P) as follows. Each haplotype *i *is represented by a node *n_i _*∈ N. For every pair of haplotypes there is an edge assigned probability *p_ij _*∈ P where *p_ij _*is the probability of IBD between haplotypes *i *and *j *at that locus.

Given a probabilistic IBD graph *G_P _*we consider a proposed IBD graph *g *= (*N, E*) over the nodes of *G_P_*. Any proposed IBD graph *g *represents a different scenario of how individuals in *G_P _*can be IBD to each other at the given genomic locus. The probability *P *(*g *| *G_P_*) of *g *conditional on the probabilistic IBD graph *G_P _*is computed as follows. We define *I *as the set of all IBD graphs derived from the nodes of *G_P_*. For each *g *∈ *I *the conditional probability of g on *G_P_*, *P*(*g *| *G_P_*) is the product of induced edge probabilities. An edge *e_ij _*= 1 if it is present in *g *and it has induced probability *p_ij_*. An edge *e_ij _*= 0 if it is not in *g *and has induced probability (1-*p_ij_*).

(1) $$P\left(g|G_{P}\right)=\underset{\forall i,j;i\neq j}{\prod }p_{ij}^{e_{ij}}{\left(1-p_{ij}\right)}^{\left(1-e_{ij}\right)}$$

### Updating the pairwise IBD graph

**Figure 1:**
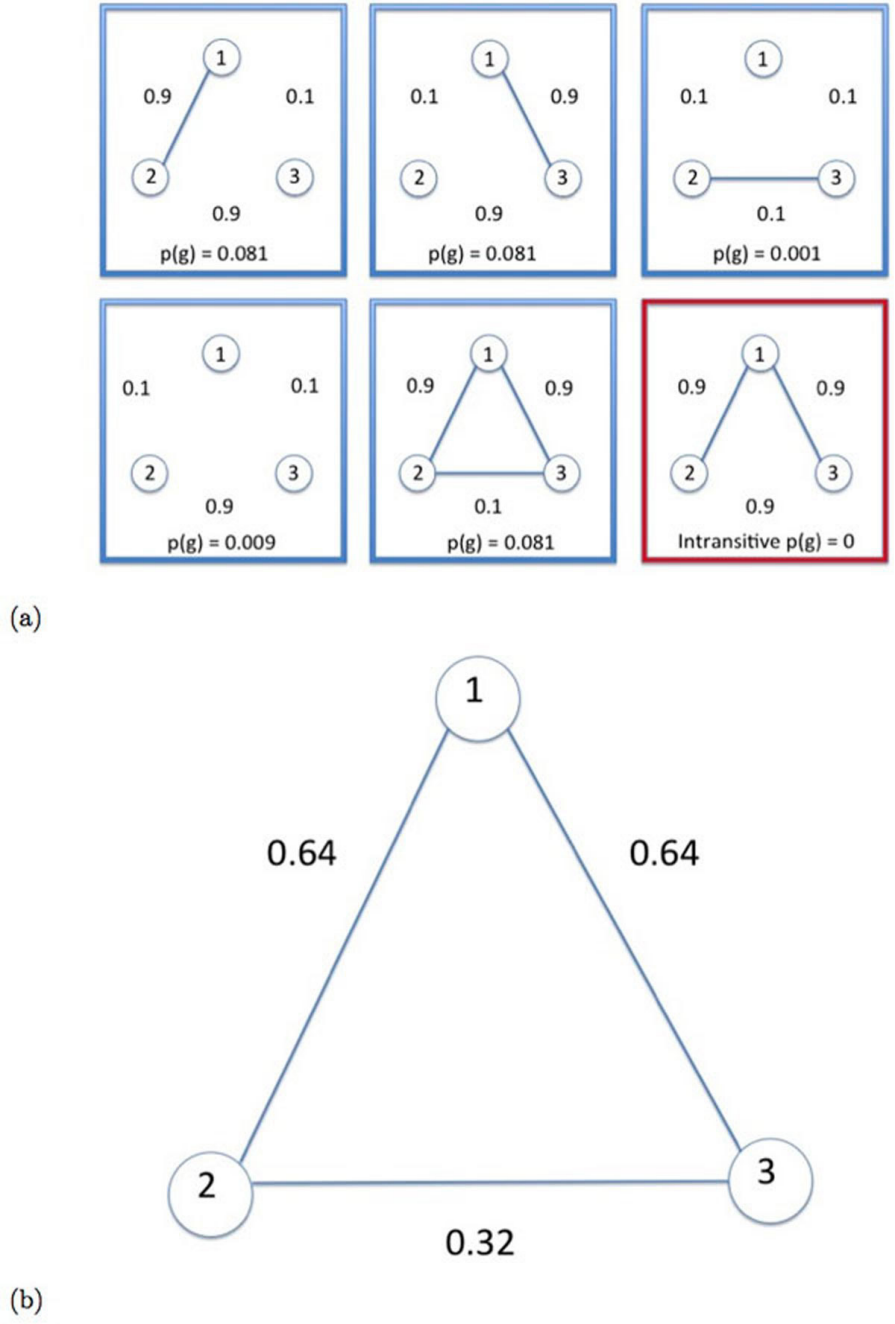
**Example IBD Graphs.**(a) Sample space of graphs and their respective probabilities. (b) Probabilistic IBD Graph with updated edge probabilities conditioned on the initial graph.

Our objective is to update the probability of each pair of individuals being IBD by conditioning on the probabilities of all pairs in the graph. The intuition is best understood with an example. Consider a probabilistic IBD graph with three nodes (*i *∈ {1, 2, 3}) and suppose the initial pairwise probabilities have been assigned by a pairwise IBD-calling algorithm such that *p*_12 _= 0.9, *p*_13 _= 0.9, and *p*_23 _= 0.1. The edges *e*_12 _and *e*_13 _have a higher probability of IBD than *e*_23_, but given that true IBD graphs obey transitivity, the probability of *e*_12 _and *e*_13 _conditioned on edge *e*_23 _will be lower. Similarly, the probability of *e*_23 _will be higher when conditioned on *e*_12 _and *e*_13 _as shown in Figure 1b. By the transitive rule when 2 of the 3 edges in a triangle have a high probability of IBD we expect the third edge to have a high probability of IBD as well.

The conditional probability of an edge given the probabilities of the graph, ${\dot{p}}_{ij}=P\left(e_{ij}=1|G_{P}\right)$, is the sum of the probabilities of all transitive graphs in which the edge is present, divided by the sum of the probability of all transitive graphs. We compute the conditional probability using only transitive graphs since we know that an intransitive graph is a biologically implausible scenario. We define *V *as the set of transitive IBD graphs derived from the nodes of *G_P_*.

(2) $${\dot{p}}_{ij}=P\left(e_{ij}=1|G_{P}\right)=\frac{\underset{g\in V;e_{ij}\in g}{\sum }P\left(g|G_{P}\right)}{\underset{g\in V}{\sum }P\left(g|G_{P}\right)}$$

All such transitive graphs and their respective probabilities are shown in Figure 1a. For illustrative purposes, we include an intransitive graph in the bottom right of Figure 1a. We update each edge by using Equation 2 and the resulting graph with updated probabilities is shown in Figure 1b. To further clarify how we compute a conditional edge probability, we compute the conditional probability of edge *e*_23_:

$$\begin{array}{c} {\dot{p}}_{23}=P\left(e_{23}=1|G_{P}\right)=\\ \frac{1\left(0.081\right)+1\left(.001\right)+0\left(.081\right)+0\left(.081\right)+0\left(.009\right)}{\left(0.081\right)+\left(.001\right)+\left(.081\right)+\left(.081\right)+\left(.009\right)}=0.324 \end{array}$$

Computing exact conditional probabilities requires computing the probability of every transitive IBD graph, which has a sample space of size O(2^*h*(*h*−1)/2^), where *h *is the number of haplotypes. Unfortunately, this is computationally infeasible to enumerate and so we develop a sampling method that can be used to efficiently approximate conditional edge probabilities.

### Efficient computation of conditional IBD probabilities

We start by generating the probabilistic IBD graph for a given genomic location. We only consider the unique positions along the genome where the IBD graph changes, or more specifically, the points where the initial IBD segments begin or end. Analyzing other positions would be redundant because the positions provide no information about how the IBD graph changes. An initial graph is generated by adding in all edges output by Refined IBD [14] that pass a LOD score threshold. Alternative pairwise IBD probability methods may be used if desired. We identify the connected components of this graph because edges that are part of disjoint components have no effect on each other when computing updated probabilities.

For each connected component *c*, we construct *G_P _*by translating the Refined IBD LOD scores to probabilities (see Section *Converting LOD scores to probabilities*). A connected component *c *may have edges that were never called by Refined IBD and thus have a probability of 0. We assign uncalled edges the probability ϵ = 0.0046 in order to ensure that *P *(*g *| *G_P_*) > 0 and that the edge can be sampled (discussed in detail in Section *Converting LOD Scores to Probabilities*). Then instead of enumerating the set of all possible valid graphs *V *inducible by *G_P _*we sample from *V*. We define *N_g _*as the current sum of probabilities of all sampled graphs so far and *N_ij _*as the current sum of probabilities of all sampled graphs containing edge *e_ij _*. At any stage in the sampling process, the estimate of the conditional probability that individuals *i *and *j *are IBD is ${\hat{p}}_{ij}=\frac{N_{ij}}{N_{g}}$. If all valid graphs are sampled with equal probability, this converges to the exact conditional probability shown in equation (2). The sampling procedure is given in Algorithm 1.

**Algorithm 1 **Graph sampling

**Input: ***G_P_*

**Output: ***N_g_*, *N_ij _*(∀*i, j*; *i *≠ *j*)

   *N_ij _*= 0, *N_g _*= 0

   **for all ***i, j ***do**

      **if ***p_ij _*≥ 0.99 **then ***e_ij _*= 1

      **else ***e_ij _*= 0

   Add edges to make all connected components of *G_p _*cliques

   Compute *P*(*g *| *G_P_*)

   *N_g _*+ = *P *(*g *| *G_P_*)

   **for all ***i, j *where *e_ij _*= 1 **do ***N_ij _*+ = *P *(*g *| *G_P_*)

   **while ${\hat{p}}_{ij}$**has not converged ∀*i, j ***do**

      Sample a random *e_ij _*and set *e_ij _*= 1 with probability *p_ij_*

      Ensure graph transitivity Compute *P *(*g *| *G_P _*)

      *N_g _*+ = *P *(*g *| *G_P _*)

      **for all ***i, j *where *e_ij _*= 1 **do ***N_ij _*+ = *P *(*g *| *G_P_*)

Edges are sampled according to a weighted distribution where weight *w_ij _*is based on *p_ij _*and is defined as:

(3) $$w_{ij}=\left\{\begin{array}{cc} \Phi \left(p_{ij}\right) & \;,\text{if}\;p_{ij}\leq 0.5\\ 1-\Phi \left(p_{ij}\right) & ,\text{otherwise} \end{array}\right)$$

(4) Φ=CDFofNormalDistribution(μ=0.5,σ=0.234)

If *σ *≈ 0, then edges with *p_ij _*≈ 1 or 0 will almost never be sampled since the selection weights of such edges will be infinitesimally small. Similarly if *σ *is too large, then all edges will be assigned similar selection weights and as a result graphs will be sampled uniformly instead of in proportion to their probability. We selected *σ *= 0.234 because it allows for efficient convergence times (see *Convergence properties and runtime*).

This weighted sampling scheme assures that edges with *p_ij _*≈ 1 or 0 are sampled less often than edges with *p_ij _*≈ 0.5. Intuitively this makes sense because we sample proposed IBD graphs in proportion to their respective *P *(*g *| *G_P_*). Edges with *p_ij _*≈ 1 induce a proposed graph with a greater *P *(*g *| *G_P _*) when they are present. Similarly edges with *p_ij _*≈ 0 induce a proposed graph with a greater *P *(*g *| *G_P_*) when they are missing. Thus, to sample the most probable graphs more often, edges with high values of *p_ij _*should typically have *e_ij _*= 1 and edges with low values of *p_ij _*should typically have *e_ij _*= 0. Changing the state of an edge can cause the proposed graph *g *to be intransitive. Therefore we add or remove edges from *g *to ensure transitivity. At each iteration if an edge has *p *≥ 0.99 then *p_ij _*is set to 1 so that we never sample very high probability edges.

**Algorithm 2 **Ensure graph transitivity

**Input: ***G_P_*, *e_ij _*that was just added or removed

**Output: **Transitive *G_P_*

   **if ***e_ij _*= 1 **then**

      Add edges to make all connected components of *G_p _*cliques

   **else**

      *S_i _*= nodes connected to *i*, *S_j _*= nodes connected to *j*

      **for all ***p_mk _*< 0.99 **do ***e_mk _*= 0

      **for **each connected component *X*, where |*X*| > 1 **do**

         ${\bar{p}}_{iX}=\frac{p_{ix}}{\left|X\right|},{\bar{p}}_{jX}=\frac{p_{jx}}{\left|X\right|}$∀ nodes *x *∈ *X*

         **if **${\bar{p}}_{iX}>{\bar{p}}_{jX}$**then **Add all nodes of *X *to *S_i_*

         **else if **${\bar{p}}_{iX}<{\bar{p}}_{jX}$**then **Add all nodes of *X *to *S_j_*

         **else **Flip a fair coin

      **for **randomly selected *k *∉ (*S_i _*∪ *S_j_*) **do**

         

$$\begin{array}{c} {\bar{p}}_{kS_{i}}=\frac{p_{ky}}{\left|S_{i}\right|},\forall y\in S_{i}\\ {\bar{p}}_{kS_{j}}=\frac{p_{kz}}{\left|S_{j}\right|},\forall z\in S_{j} \end{array}$$

         **if ${\bar{p}}_{kS_{i}}>{\bar{p}}_{kS_{j}}$ then **Add *k *to *S_i_*

         **else if ${\bar{p}}_{kS_{i}}<{\bar{p}}_{kS_{j}}$ then **Add *k *to *S_j_*

         **else **Flip a fair coin

      Make *S_i _*and *S_j _*cliques

We continue selecting edges until we reach a convergence criterion or a user-set time limit has been reached. As a convergence criterion we check if all edge probabilities change less than 1 × 10^−11 ^for 5000 sequential iterations. With larger graphs that may never reach the convergence criteria, we allow the user to set a runtime limit. After convergence or hitting the user runtime limit, we output all edges and their respective updated probabilities. We tried a variety of sampling schemes to explore the space of graphs and selected this one due to its performance over simulated datasets (see *Application to simulated data*).

### Converting LOD scores to probabilities

**Figure 2:**
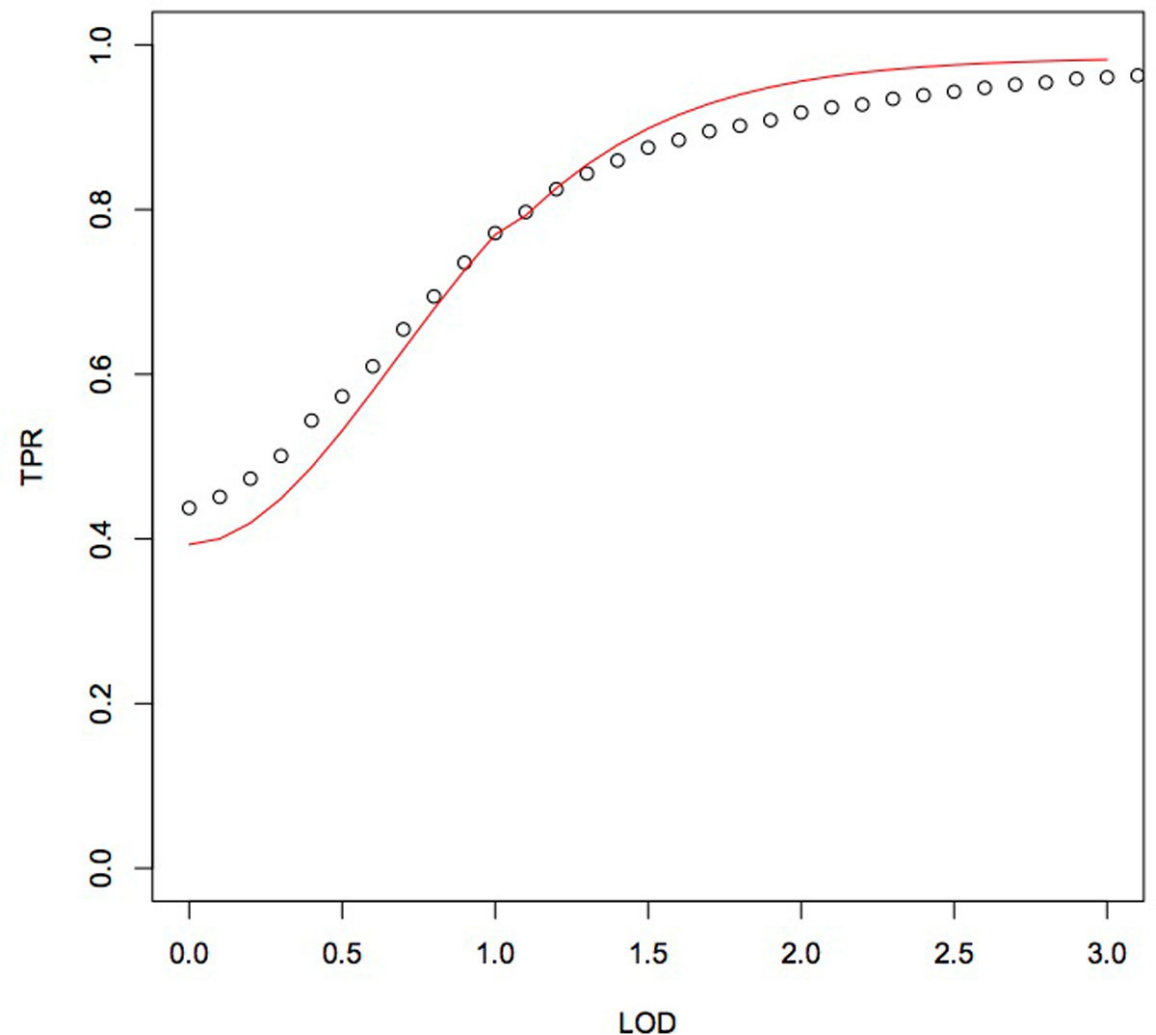
**Refined IBD true positive rates as a function of LOD score.** Refined IBD true positive rates as a function of LOD score are shown as black dots and our converted probabilities as a function of LOD score is shown as a red line.

To find the relationship between LOD scores and the true positive rate of IBD segments we ran Refined IBD on simulated data (see *Application to simulated data*) using a LOD score cutoff of 0.1 and a length cutoff of 0.1 centimorgans. A true positive segment is defined as a predicted segment that is at least 50% true IBD. We fit a curve to the observed relationship between LOD score and true positive rate of IBD segments (see Figure 2). The equation of our curve is of the form *p *= (2*o *+ *af*)/(*o *+ *f *) where *o *= posterior odds, *f *= (prior * (10^3^)/.997)−(prior * (10^3^)), *a *= (1 − LOD)^3^/7 if LOD ≤ 1, and *a *= −0.15 otherwise. The values for *f *and *a *were chosen to maximize the fit of the curve.

Since the Refined IBD [14] LOD score is the negative base 10 log likelihood of one shared haplotype divided by the likelihood of no shared haplotypes, we use Bayes rule for odds to convert a LOD score into a posterior odds: $O\left(A|B\right)=O\left(A\right)*\frac{L\left(A|B\right)}{L\left(A^{c}|B\right)}$, where *O*(*A *| *B*) is the posterior odds, *O*(*A*) is the prior odds, and $\frac{L\left(A|B\right)}{L\left(A^{c}|B\right)}$ is the likelihood ratio.

For the prior, we use the probability of any two individuals in the sample being IBD at any point in the genome, *ϵ *= 0.0046. This is the average proportion of the genome shared IBD between all pairs of individuals estimated using results from Refined IBD over simulated data. For edges that have a probability of 0 (i.e. an edge with no pairwise call), we assign a probability equal to the prior because otherwise these edges would never be sampled and graphs would have a probability of 0.

Ideally, the relationship between LOD score and true positive rate is given by *p *= *odds*/(1 + *odds*). However, the relationship between LOD score and true positive rate in our sample of simulated individuals deviates from this theoretical relationship. Our function and *p *= *odds*/(1 + *odds*) are of the same form (i.e. *g*(*x*) = (*cx *+ *d*)/(*mx *+ *n*)). This served as our motivation in defining the conversion function. Lastly, given that the simulated data we generated is reflective of European population growth, the relationship between LOD score and true positive rate may differ in other populations (see Discussion).

### Merging results across graphs and inferring new segments

IBD segments can span multiple regions and our method analyzes IBD at a single region. The probability of IBD between two individuals can therefore be output at multiple adjacent regions by our method. Furthermore, the IBD probability may be assigned a different value in each region due to the inexact nature of the sampling method. If the same IBD segment is assigned different probabilities across multiple loci we use the maximum value across all regions.

Once an IBD graph is analyzed using the sampling procedure, edges that were previously missing (i.e. those that were not called by Refined IBD) are output with a start and stop site that is equal to the intersection of all IBD segment boundaries in the graph. Since we do not look in the region for sequence identity between haplotypes we can only output the probability that IBD exists somewhere within the region. These new segments may also overlap with other called IBD segments. In order to reconcile overlapping IBD segments, we merge them provided that they pass a probability threshold set by the user and that they lie on the same haplotype. As the final probability, we use the maximum ${\hat{p}}_{ij}$ of the merged segments. For all analyses presented here, we only merged segments that had a probability of 0.99 or greater.

### Creating simulated IBD data

We generated simulated genotype data as previously described by [14]. To start, we use Fastsimcoal [21] to generate phase known DNA sequence data of 2000 diploid individuals. A single individual is represented as one chromosome consisting of ten independent 30 MB regions, each with a mutation rate of 2.5 × 10^−8 ^and a recombination rate of 10^−8^. The population simulated begins with an effective population size of 3000 diploid individuals with a growth rate of 1.8% at time t = 300 (where t is the number of generations ago from the present). Moving forward in time, the growth rate was changed to 5% and to 25% at times t = 50 and t = 10 respectively, resulting in a final effective populations size of 24,000,000 at t = 0. The simulation is reflective of European population sizes estimated from the linkage disequilibrium of common variants [22].

Using the DNA sequence data we create genotype data by first filtering single nucleotide polymorphisms (SNPs) that were not bi-allelic with a minor allele frequency (MAF) less than 2%. Next, we choose 10,000 variants uniformly by MAF (where 2% ≤ MAF ≤ 50%) per 30 MB region. This SNP density is in line with that of a 1,000,000 SNP genotyping array. Finally, we remove all phase information and apply a genotyping error at a rate of .05% by turning heterozygous genotypes into homozygous genotypes and vice versa. Using the simulated genotype data, we use Refined IBD [14] to phase the data and call pairwise IBD. We define true IBD segments as those segments longer than or equal to 0.1 centimorgan. A potential consequence of this approach to creating simulated data is that the resulting IBD graph may not completely obey transitivity.

## Results

### Convergence properties and runtime

We first verify that the conditional probabilities estimated from our sampling approach, ${\hat{p}}_{ij}$, converge to the true edge conditional probabilities, ${\dot{p}}_{ij}$. We randomly create three to eight node probabilistic IBD graphs with edge probabilities drawn uniformly from the open interval (0, 0.99). For each graph, we enumerated every transitive IBD graph to compute the exact conditional edge probability ${\dot{p}}_{ij}$. It is computational infeasible to compute exact probabilities for graphs larger than 8 nodes since all transitive graphs must be enumerated. We then ran our sampling approach over each graph and at each iteration *l*, we calculated the average percent difference between ${\hat{p}}_{ij}$ and ${\dot{p}}_{ij}$, which we call *δ_l_*.

$$\begin{array}{c} \delta_{l}=\underset{\forall i\neq j}{\sum }\frac{\left|p_{ij}-{\hat{p}}_{ij}^{l}\right|}{p_{ij}}\\ {\hat{p}}_{ij}^{l}=\text{conditional}\;\text{edge}\;\text{probability}\;\text{at}\;\text{iteration}\;l \end{array}$$

**Figure 3:**
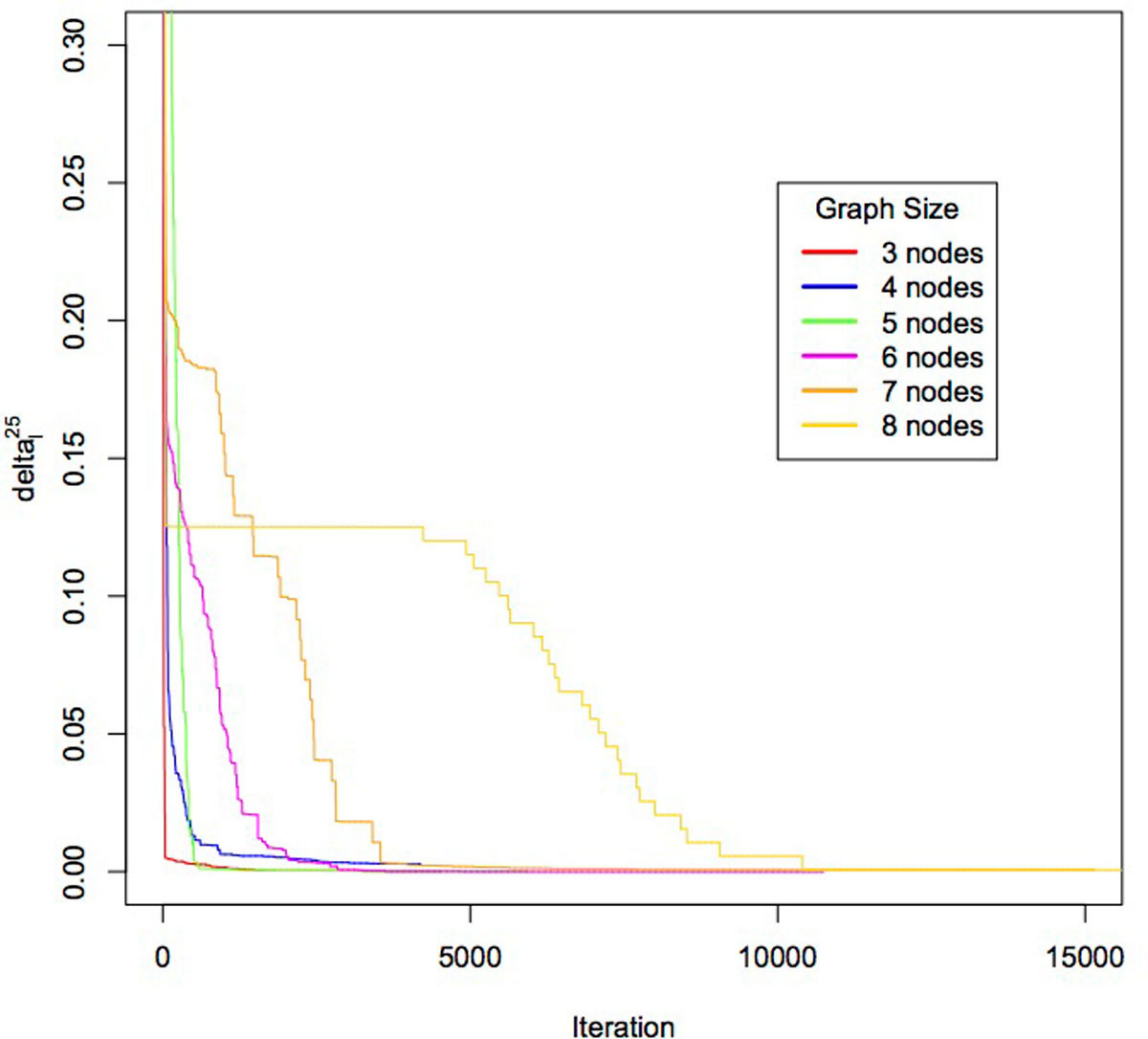
**Iterations needed for convergence.** On the x-Axis is the number of iterations and on the y-axis is the value of $\delta_{l}^{25}$ which is the average percentage edge delta over 25 runs.

We ran PIGS 25 times and calculated $\delta_{l}^{25}$ which is *δ_l _*averaged over all 25 runs. From Figure 3 we see that for graphs with 3 to 7 nodes, edges are within 1% of true conditional probability after 5000 iterations. For 8 node graphs, the probabilities are within 15% of the true ${\dot{p}}_{ij}$ after 5000 iterations and within 5% within 7500 iterations. We recorded the average runtime of the 25 runs and show the results in (Table 1). While it is computationally feasible to sample until convergence for small graphs, this approach will not scale to genome-wide IBD studies of a large number of individuals. Instead PIGS takes as input a user specified time limit for sampling each region.

**Table 1:** Average Runtime of different sized graphs over 25 iterations.

Nodes	Time (CPU seconds)
3	1.34
4	2.79
5	6.31
6	17.33
7	46.99
8	80.19

### Application to simulated data

Ultimately, the metrics of merit are the IBD calls themselves, not IBD probabilities. IBD calls can be made from IBD probabilities using a thresholding approach in which all probabilities exceeding a threshold are output as IBD. Alternatively, methods such as DASH [12], EMI [19], and IBD-Groupon [18] leverage the clique nature of IBD graphs to output cliques over a region as opposed to IBD pairs. The choice of IBD calling method is a function of the objective of the study. For example, DASH was designed specifically for association testing in which individuals in a clique are given a psuedo-genotype of 1 and all others are given a pseudo-genotype of 0. Other testing methods examine the distribution of IBD between cases and controls [13,9,10] and rely on IBD calls that powerfully and accurately cover true IBD segments. For population genetics purposes such as inferring demographic history [5], the distribution of IBD segments sizes is the figure of merit.

This diversity of uses of IBD precludes any single metric as being the gold standard for assessing the quality of IBD calls. Therefore, we compare several different methods of computing IBD probabilities and calling IBD over a range of metrics. We compare a thresholding approach to calling IBD applied to PIGS probabilities as well as Refined IBD LOD scores. We also examine the behavior of the clique-calling approaches DASH [12] and EMI [19] when applied to Refined IBD output and PIGS output. We attempted to include IBD-Groupon but in its current implementation some hard-coded parameters make it unsuitable for the sample sizes we examined here. This will be addressed in a future release (personal communication with Dan He).

We created simulated genotype data on ten 30 MB regions for 2000 individuals (see *Creating simulated IBD data*). We generated IBD calls from Refined IBD by using a LOD threshold of 3. For PIGS, we first generated pairwise graphs from Refined IBD by using a LOD threshold of 0.1 and a segment length cutoff of 0.1 centimorgans. PIGS was then run over the pairwise graphs for a maximum of 2 minutes and IBD calls were made using a probability threshold of 0.99. IBD calls for Germline were generated using their suggested parameters "-haploid -bin out -min_m 1 -bits 32 -err_hom 1 -err_het 1" [17] after phasing genotype data using fastIBD [11]. DASH was run over the Refined IBD calls passing a LOD threshold of 3. All results were filtered to have a minimum segment length of 0.5 centimorgans.

#### Identification of IBD segments

**Figure 4:**
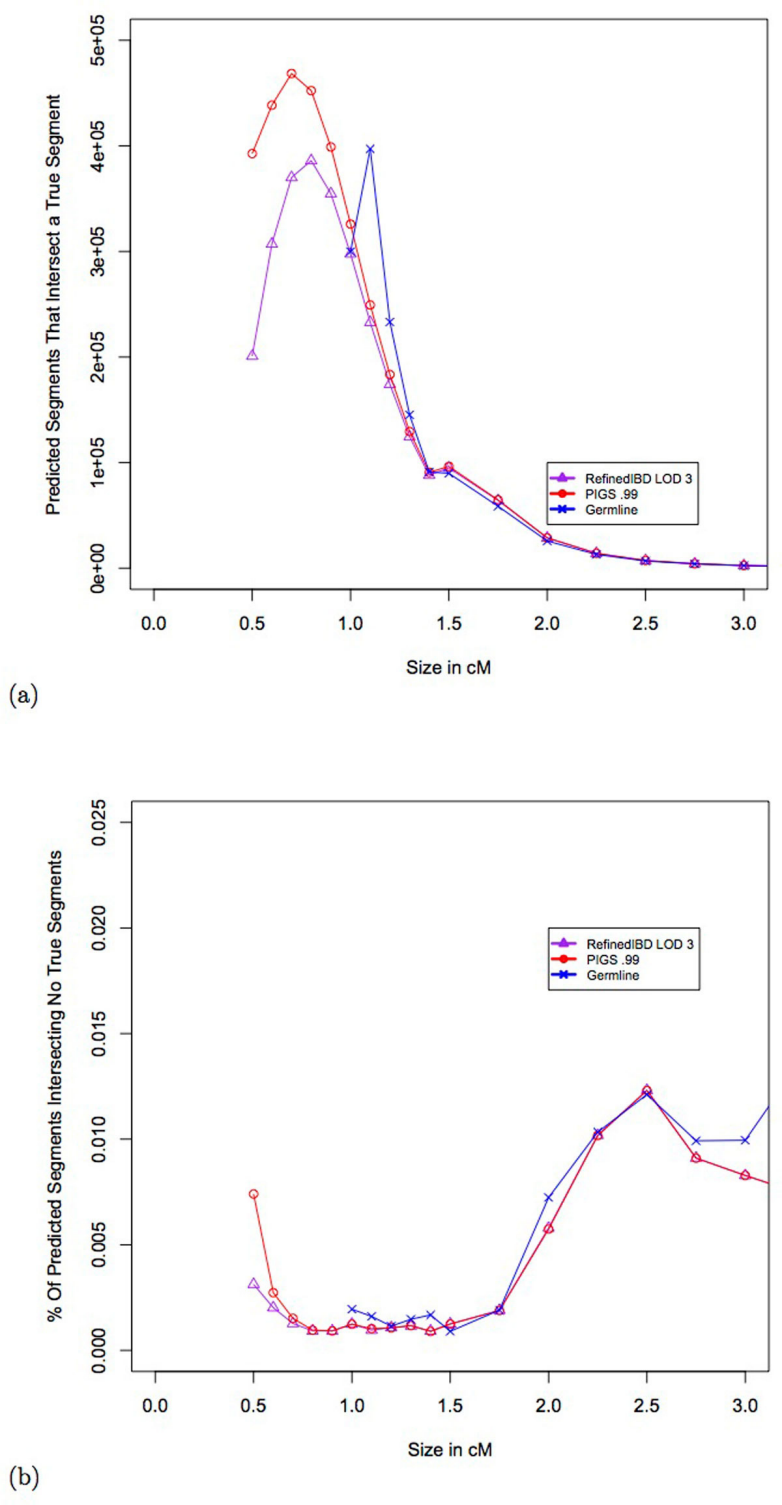
**Power and error rates as a function of IBD segment counts.** (a)Number of predicted segments overlapping a true IBD segment is shown on the y-axis. The x-axis shows the size of the predicted segment in centimorgans. (b)The percentage of predicted segments that have no overlap with a true segment is shown on the y-axis. The x-axis shows the size of the predicted segment in centimorgans.

For a given genomic locus, the power of tests comparing the distribution of IBD in cases or between cases and controls [13,10,9], is a function of the number of true IBD segments intersected by predicted segments. We therefore performed an analysis of the total number of true IBD segments intersected by IBD calls from Refined IBD, Germline, and PIGS. The results shown in Figure 4a show that PIGS substantially outperforms Refined IBD for small IBD segments. DASH was not included in this analysis because it was not designed for this purpose and the resulting error rates were 10 fold higher than PIGS and Refined IBD even at 1 centimorgan segments. For predicted segments of size 0.5, 0.6, 0.7, 0.8, 0.9, and 1 centimorgans, there was an increase of 95%, 43%, 27%, 17%, 12%, and 9% in the number of predicted segments intersecting a true segment over Refined IBD. For predicted segments of size 1.1, 1.2, and 1.3 centimorgans, Germline was able to detect 60%, 27%, and 12% more segments than PIGS but the calls were less accurate (See *Accuracy of IBD segments*).

In order to assess the error rate we examined the fraction of segments that did not intersect any true IBD segment. Note that this is error rate may be inflated due to the fact that true segments are required to be at least 0.1 centimorgans (see *Creating simulated IBD data*). The results shown in Figure 4b demonstrate that PIGS has nearly identical error rates to Refined IBD at small segments. However, at 0.5 centimorgans the error rate increases from 0.3% to 0.7%; this is a modest increase relative to the 95% increase in the number of segments identified. Germline, in general, was similar to the other methods in terms of error rates for segments between 1 and 2 centimorgans.

#### Accuracy of IBD segments

**Figure 5:**
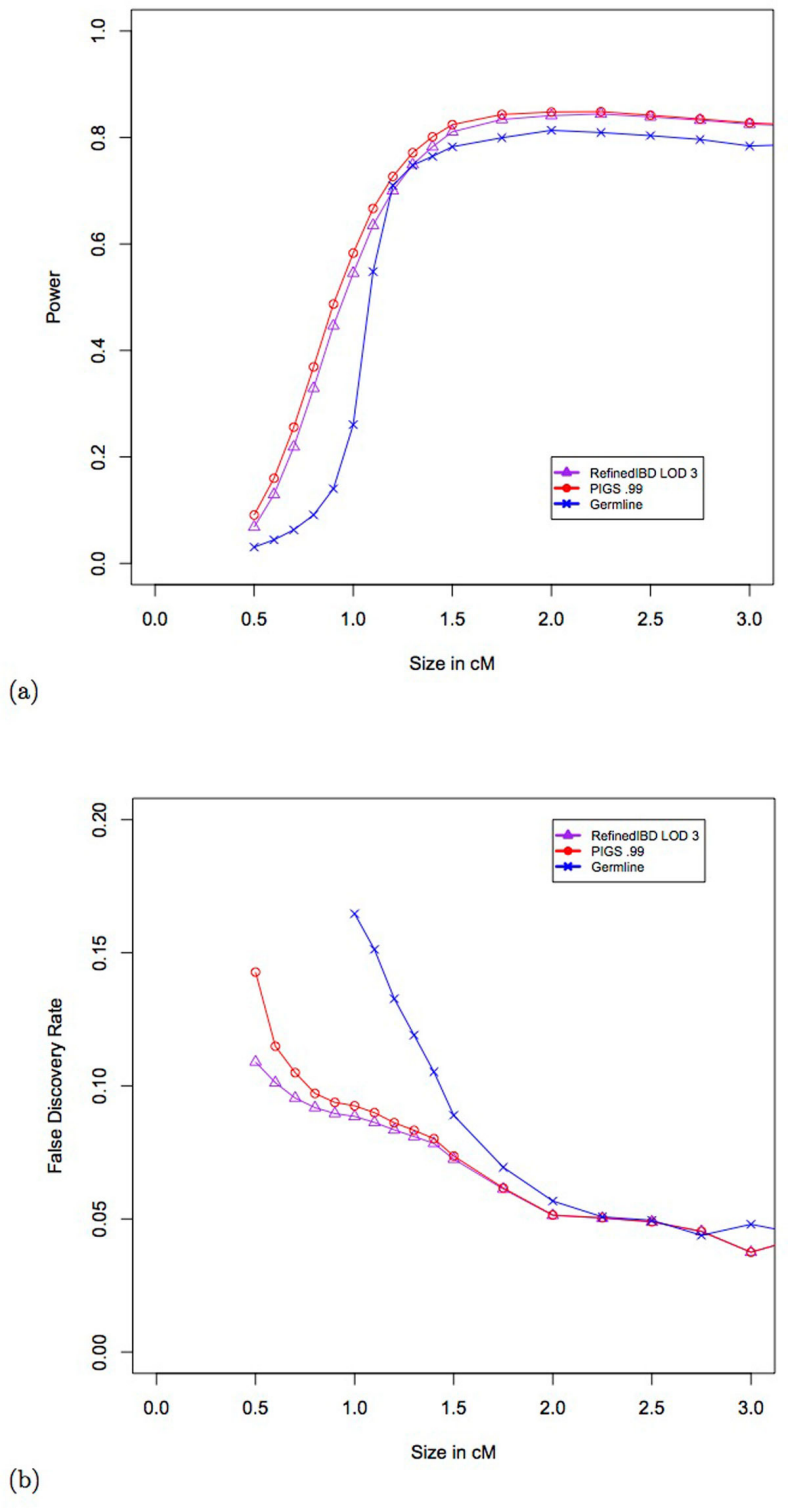
**Power and error rates as a function of IBD segment proportions.** (a)The average proportion of a true segment detected is shown on the y-axis. The x-axis shows the size of the true segment in centimorgans. (b)The average proportion of predicted segments that do not intersect any true IBD segment is shown on the y-axis. The x-axis shows the size of the predicted segment in centimorgans.

In population genetics settings, such as inferring demography [23,5], methods often rely on the distribution of IBD segment lengths. The figure of merit here is related to the accuracy of predicted segments recovered. We first examined power, the average proportion of true IBD segments that were overlapped by predicted segments. For true segments between 0.5 and 2.5 centimorgans our method had modestly greater power (Figure 5a). This came at the expense of a slight increase in false discovery rate (FDR) as shown in Figure 5b. The false discovery rate is defined as the average proportion of predicted segments that does not overlap a true IBD segment. This is somewhat expected since new segments from PIGS use existing IBD segment boundaries to approximate the new start and stop sites. The greatest decrease comes at 0.5 centimorgans where PIGS predicts 95% more segments than Refined IBD. However, the difference in FDR between Refined IBD and PIGS is still less than 5% (10% versus 14%). On the other hand, for segments of size between 0.6 and 1.5 centimorgans, PIGS predicts 23% more segments while keeping the FDR within 1% of Refined IBD.

**Figure 6:**
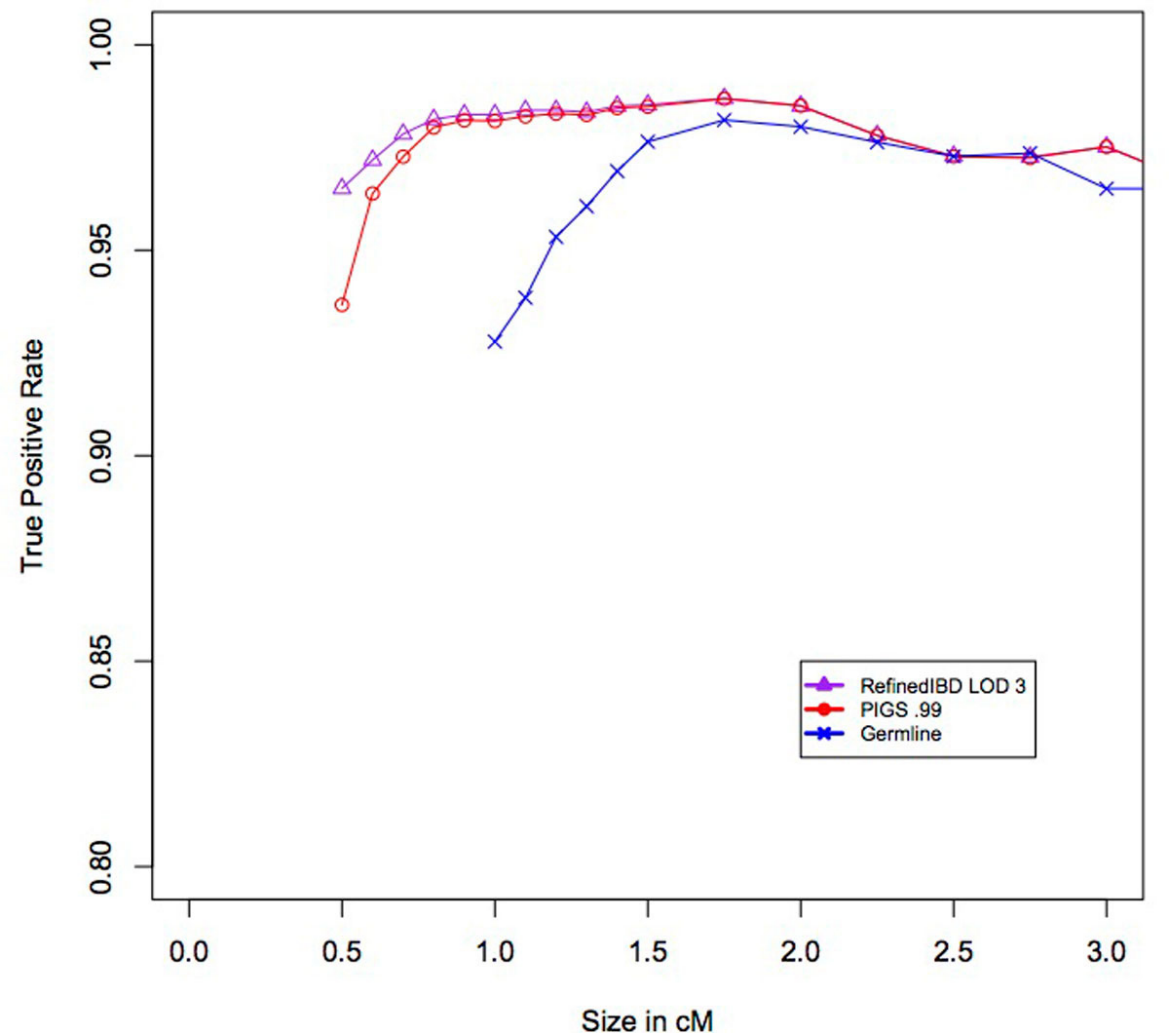
**True positive rate.**The percentage of predicted segments that overlaps at least 50% with a true IBD segment is shown on the y-axis. The x-axis shows the size of the predicted IBD segment in centimorgans.

We also examined the true positive rate, defined as the percentage of predicted segments with at least 50% overlap with a true segment. Compared to Refined IBD the true positive rate for PIGS drops slightly for segments that are smaller than 1 centimorgan but the difference is less than 1% for all sizes except for at 0.5 centimorgans where it is 3% (Figure 6). The reason for this drop in performance is at least partly due to the fact that we add new segments according to the IBD graph without specifically examining the sequence. Given the results of the previous section, the most likely explanation is that the PIGS predicted segments intersect true IBD segments, but not at the 50% threshold required by definition of a true positive. Based on these results PIGS could be used for population genetics purposes, but users should take into account the slight increase in error rates for smaller segment sizes.

#### Identification of cliques

In a genome wide association study (GWAS) association tests are typically performed on individual single nucleotide polymorphisms. Cliques of IBD segments can also be leveraged to increase power in association tests [12,24]. In this setting IBD serves as a representation of SNPs not contained on the genotyping platform, and the figure of merit is how well the true IBD cliques are captured by predicted IBD segments.

For 10000 random positions along the genome, we created predicted IBD graphs for Refined IBD, PIGS, P-DASH, P-EMI (DASH and EMI using PIGS as input), R-DASH, and R-EMI (DASH and EMI using Refined IBD as input). For Refined IBD and PIGS, all segments of size 0.5 centimorgans or greater were used if they passed a LOD threshold of 3 and probability threshold of 0.99 respectively. DASH and EMI are both algorithms that create cliques in a given window. DASH starts with the biggest connected component and creates dense subgraphs by cutting out false-positive edges. EMI on the other hand starts with seed subgraphs and adds edges that it believes to be true IBD. For DASH we used default parameters "-win 500000 -density 0.6 -r2 0.85 -min 4". For EMI we used the parameters "-win bp 200000 -den 0.6 -min 3 -wgt bp 100000 1000000". However with these EMI parameters, R-EMI had an error rate 3 to 8 times greater than the other methods depending on the size of the clique. Instead we used the weight parameter "-wgt 7th 3 40" for Refined IBD input to EMI which uses the LOD score instead of the length to weight the edges and improved performance.

At each position we examined cliques in the true graph that overlapped with a connected component in the predicted graph for any method. The true graph was generated with all true IBD calls regardless of size and all connected components were converted to cliques. Table 2 shows the power of each method to detect an edge of a true clique of a given size. The power here is defined as the average proportion of edges in a true clique that are called correctly by a given method. This is not the power to recover an entire clique, but an estimate of the number of edges in a clique that are recovered. At any clique size, PIGS detects a higher proportion of edges than Refined IBD. For P-DASH and R-DASH the power of both methods are very similar with P-DASH only showing a very modest increase in power depending on the clique size. However, when comparing P-EMI and R-EMI we see 2-5% increases in power for P-EMI. To verify that the gain in power for P-DASH and P-EMI was due to PIGS and not due to leveraging clique information twice, we used EMI and DASH output as input into a second round of EMI and DASH. We observed virtually no change in power or error rate showing that PIGS is providing the increase in performance. All methods lacked power when considering very large cliques and this is most likely due to the fact that very large cliques are generated from small segments of IBD (i.e. < 0.5 centimorgan).

**Table 2:** The power of each method to detect a true clique of a given size.

Bin	R-DASH	R-EMI	R-IBD	P-DASH	P-EMI	PIGS
120-149	0.08	0.09	0.02	0.09	0.11	0.11

90-119	0.11	0.13	0.02	0.11	0.15	0.14

60-89	0.16	0.20	0.05	0.17	0.23	0.16

30-59	0.24	0.33	0.10	0.25	0.38	0.23

0-29	0.38	0.49	0.21	0.38	0.52	0.34

We also assessed the false positive rate of each clique-based method. The false positive rate was defined as the average proportion of predicted edges that are not part of a true clique. Table 3 shows the false positive rate of each method for a given size of a predicted clique. The false positive rate of PIGS is slightly higher than Refined IBD for most clique sizes, but the increase in false positive rate is modest (within 2%) for all clique sizes. As was the case with power, the error rates of P-DASH and R-DASH are nearly identical. The biggest difference is for cliques with 60-89 nodes, where P-DASH has a 4% higher false positive rate. We see similar behavior for R-EMI and P-EMI, where for cliques of size 90-119 nodes, the error rate goes from 5% to 9%. Based on these results we recommend using EMI to perform clique calling on PIGS output as it provides lower error rates and higher power than DASH.

**Table 3:** The false positive rate of each method when detecting a true clique of a given size.

Bin	R-DASH	R-EMI	R-IBD	P-DASH	P-EMI	PIGS
120-149	NA	0.05	0.02	0.11	0.05	0.02

90-119	0.09	0.05	0.02	0.09	0.09	0.03

60-89	0.03	0.04	0.01	0.07	0.04	0.03

30-59	0.02	0.03	0.01	0.02	0.04	0.02

0-29	0.01	0.03	0.01	0.02	0.03	0.01

### Application to real data

#### Identification of IBD segments

We applied PIGS, RefinedIBD, DASH, and EMI to 489 Latino trios from the Genetics of Asthma in Latino Americans (GALA) cohort [25]. The availability of trio genotype data allows us to phase the genotypes with high accuracy by taking into account the rules of Mendelian segregation. The increased phasing accuracy in turn boosts the power to detect IBD segments because phasing errors are a major source of difficulty in calling IBD [26]. To evaluate how well a given method is able to identify segments of IBD in real data we used IBD segment calls made using Refined IBD in a trio-aware mode (trio-IBD segments). Trio-IBD segments were thresholded at a length of 0.1 centimorgans and at a LOD score of 3. We then asked how many IBD calls made by a given method without access to the near-perfect trio phasing overlapped with trio-IBD segments. DASH and EMI were run in the same way as we described for identifying cliques (using PIGS and RefinedIBD as input), however the resulting clique edges were converted into IBD calls and merged with the original input. We include the clique calls here as IBD calls because we do not know the structure of the real IBD graph. All IBD calls were thresholded at a segment size of 0.5 centimorgans.

**Figure 7:**
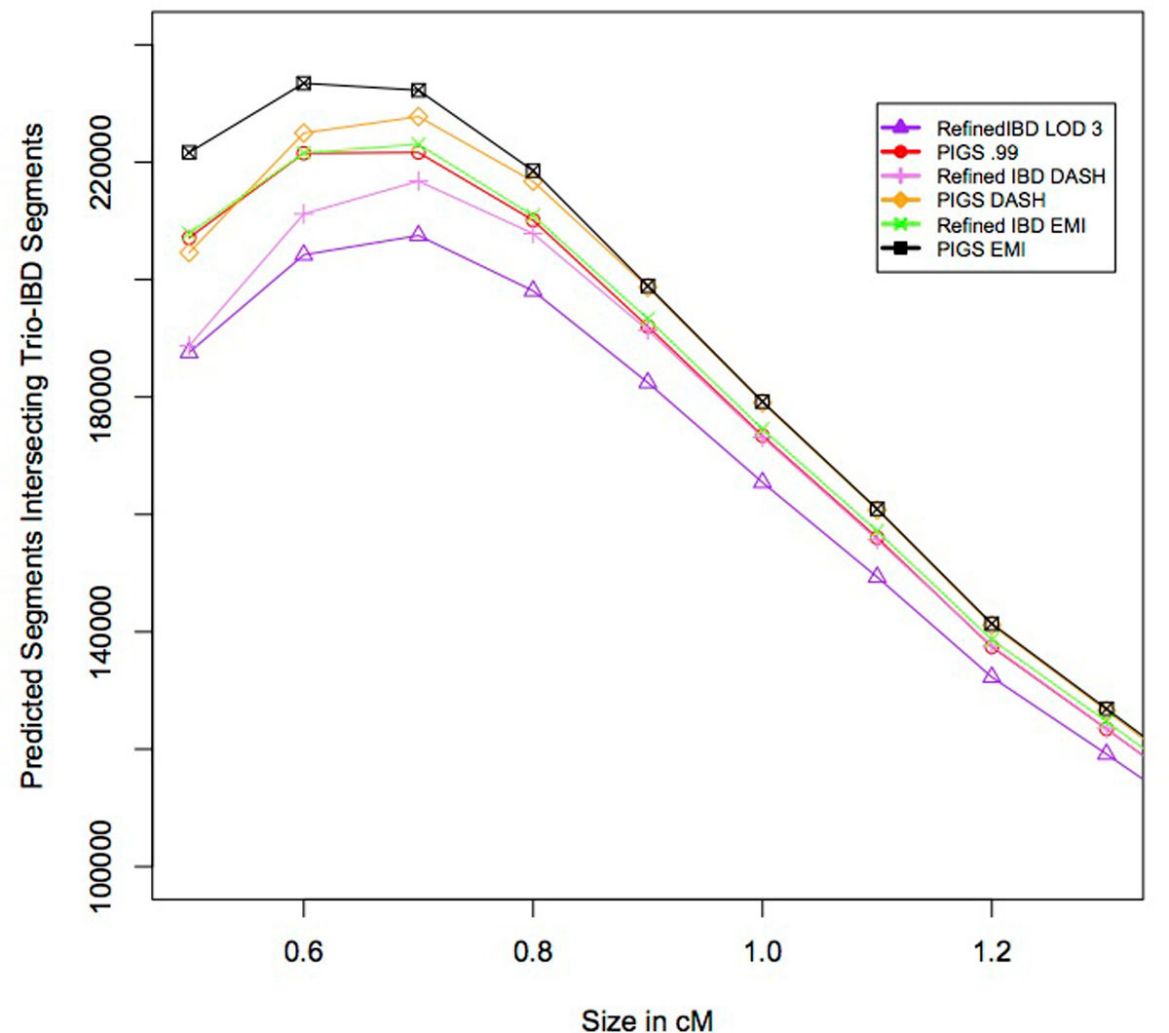
**Number of trio-IBD segments identified.** The number of predicted segments that overlaps a trio-IBD segment is shown on the y-axis. The x-axis shows the size of the predicted IBD segment in centimorgans.

As shown in Figure 7, when considering PIGS and Refined IBD calls at 0.5 centimorgans, there is an increase of 10% in the number of segments identified by PIGS over Refined IBD. After applying DASH and EMI to the input of both methods we see an increase of 8% and 7%, respectively, for PIGS input. It is clear that both DASH and EMI improve the power of both main approaches to detect IBD for use in association studies regardless of the segment size. DASH and EMI seem to perform similarly in terms of boosting power when called segments are bigger than 0.8 centimorgans, but EMI appears to have the upper hand for anything smaller. For example, at 0.5 centimorgans the difference between EMI and DASH for PIGS input is 8% but at 0.8 centimorgans the difference is only 0.8%. Across all segment sizes, we see increases of 4%, 3%, and 2.5% for PIGS, P-DASH, and P-EMI over their Refined IBD counterparts. The increases are more modest than in the simulated data, most likely due to the fact that without sequencing data we are underpowered to detect small segments of IBD even when trio phased genotypes are available.

PIGS and Refined IBD called 3134591 and 2968480 segments that overlapped with at least one of trio-IBD segment, respectively, which equates to a 6% increase. Similar increases are also seen when using PIGS input to DASH and EMI, with a 4% increase (3177234 versus 3047734) for DASH and a 3% increase (3263594 versus 3158818) for EMI. 1330207 PIGS and 803527 Refined IBD calls did not overlap with any trio-IBD segments. Because we only have access to true positives in the real data, there is no perfect way to determine the false positive rate of any of these methods, and it could be argued that PIGS increases the power to detect IBD at the expense of a higher false positive rate. To determine if this was the case, we made random IBD calls along the genome. As an example consider the calls of size 0.5 centimorgans, where PIGS made 349221 calls and Refined IBD made 262064 calls, a difference of 87157 calls. Of these, 207122 PIGS and 187582 Refined IBD segments overlap a trio-IBD segment, which is an increase of 19540 segments. After making 87157 random calls (of length 0.5 centimorgans) we only identified 453 segments compared to the 19540 we observed originally. This means that even if Refined IBD were to make an additional 87157 random guesses along the genome we would not expect Refined IBD to have the same power as PIGS, showing the increase in performance is not entirely due to false positives. Furthermore, if we assumed all non-overlapping segments are false positives both Refined IBD and PIGS have an error rate over 20%, which is not reflective of the simulations where the error rate for both methods was below 1% (see Figure 4b) [14].

Given these results, we conclude that the increased performance in PIGS was not driven by the extra IBD calls and that the majority of the non-overlapping segments are indeed true as suggested by the simulation results. Assuming that the true false positive rate in real data is similar to simulation data, the large increase in predicted segments that overlap trio-IBD segments when using PIGS (with or without a clique calling method) shows the potential for substantial power increases when using PIGS for IBD mapping studies.

## Conclusion

We have developed a new efficient method (PIGS) for simultaneously computing the probability of IBD between multiple haplotypes at a genomic region. PIGS combines the computational efficiency of pairwise methods with the power advantages of multiway methods. We demonstrated that PIGS converges to the correct probabilities of conditional IBD probabilities for small IBD graphs. For IBD graphs with both small and large numbers of individuals we showed that the approximate probabilities from PIGS produce a substantial improvement in the power to identify small IBD segments and recover IBD edges from cliques relative to previous approaches.

PIGS relies on accurate pairwise probabilities in order to compute conditional probabilities. In this work we scaled the probabilities according to the results of simulated segments of IBD. This has been the approach of previous methods [10,18,14,12,15], as there is currently no mechanism for assessing true probabilities in real data. This approach is not guaranteed to be accurate for all populations. If the demographic history of the population of interest is substantially different from the one simulated here, additional simulations should be done to assess the relationship between LOD scores and probability of IBD.

In some scenarios, such as the inference of demographic history [5], the metric of merit is not the power to identify segments, but the accuracy of the distribution of IBD segment lengths. Because PIGS does not currently utilize genotype or sequence data to refine newly identified IBD segments it is not as accurate as Refined IBD for small segments. One possible future approach is to use powerful, but computationally expensive multiway IBD calling methods such as the MCMC proposed by Moltke *et al*. 2011 [15] to examine the new regions identified from PIGS.

In our analysis here we restricted our analysis to segments that were at least 0.5 centimorgans in size. There may be IBD segments that are much smaller in size (<< 1 centimorgan) and methods such as HapFABIA [7] are able to identify these small segments. HapFABIA uses an efficient bi-clustering approach but relies on the existence of rare variation in the data. Given that we did not have sequencing data available, we did not compare our method to HapFABIA. However in the future, sequencing data will be more readily available and we hope to see how if PIGS can also be leveraged to improve the power existing methods.

Clique-calling methods such as DASH, EMI, and IBD-Groupon use IBD probabilities such as those output from PIGS and Refined IBD to identify cliques of IBD segments. Clique-calling methods are typically used to increase the power of IBD mapping studies. We showed that these methods can substantially increase the power to detect the edges of IBD graphs. The exact relationship between the power of a given IBD mapping approach and the number of edges discovered remains to be shown. Going forward, having a better grasp of how power and false positive rates of predicted graphs affect IBD mapping methods will be important to maximize the utility of clique based mapping approaches.

The current sampling scheme for PIGS was selected for its performance in identifying IBD segments. There are many different methods of exploring the space of transitive graphs. Our focus in this work was medical genetics, but alternative sampling schemes could be explored to optimize segment accuracy instead of power to detect segments. Given the substantial improvement in the number of identified IBD segments of our method, we expect that PIGS will facilitate improvements in IBD based disease association studies and provide new inroads into identifying small segments of IBD.

## Competing interests

The authors declare that they have no competing interests.

## Authors' contributions

D.S.P., N.Z., Y.B., F.H. designed the methods and experiments. D.S.P. implemented experiments and software. C.E., D.G.T., and E.G.B. provided data, D.S.P. and N.Z. wrote the manuscript.

## Declarations

Publication charges for this work were partly funded by Grant Number: 1K25HL121295-01A1 to N.Z., and partly funded by the RWJF Amos Medical Faculty Development Award and the American Asthma Foundation to E.G.B.

This article has been published as part of *BMC Bioinformatics *Volume 16 Supplement 5, 2015: Selected articles from the 10th International Symposium on Bioinformatics Research and Applications (ISBRA-14): Bioinformatics. The full contents of the supplement are available online at http://www.biomedcentral.com/bmcbioinformatics/supplements/16/S5.
